# Health economic analysis of the integrated cognitive assessment tool to aid dementia diagnosis in the United Kingdom

**DOI:** 10.3389/fpubh.2023.1240901

**Published:** 2023-09-29

**Authors:** Judith Shore, Chris Kalafatis, Angela Stainthorpe, Mohammad Hadi Modarres, Seyed-Mahdi Khaligh-Razavi

**Affiliations:** ^1^York Health Economics Consortium, University of York, York, United Kingdom; ^2^Cognetivity Ltd., London, United Kingdom; ^3^Department of Old Age Psychiatry, South London and Maudsley NHS Foundation Trust, King’s College London, London, United Kingdom; ^4^Department of Stem Cells and Developmental Biology, Cell Science Research Centre, Royan Institute for Stem Cell Biology and Technology, ACECR, Tehran, Iran

**Keywords:** ICA, CognICA, dementia, cognitive screening, AI, National Health Service (NHS), health economics

## Abstract

**Objectives:**

The aim of this study was to develop a comprehensive economic evaluation of the integrated cognitive assessment (ICA) tool compared with standard cognitive tests when used for dementia screening in primary care and for initial patient triage in memory clinics.

**Methods:**

ICA was compared with standard of care comprising a mixture of cognitive assessment tools over a lifetime horizon and employing the UK health and social care perspective. The model combined a decision tree to capture the initial outcomes of the cognitive testing with a Markov structure that estimated long-term outcomes of people with dementia. Quality of life outcomes were quantified using quality-adjusted life years (QALYs), and the economic benefits were assessed using net monetary benefit (NMB). Both costs and QALYs were discounted at 3.5% *per annum* and cost-effectiveness was assessed using a threshold of £20,000 per QALY gained.

**Results:**

ICA dominated standard cognitive assessment tools in both the primary care and memory clinic settings. Introduction of the ICA tool was estimated to result in a lifetime cost saving of approximately £123 and £226 per person in primary care and memory clinics, respectively. QALY gains associated with early diagnosis were modest (0.0016 in primary care and 0.0027 in memory clinic). The net monetary benefit (NMB) of ICA introduction was estimated at £154 in primary care and £281 in the memory clinic settings.

**Conclusion:**

Introduction of ICA as a tool to screen primary care patients for dementia and perform initial triage in memory clinics could be cost saving to the UK public health and social care payer.

## Introduction

Dementia is defined as an acquired loss of cognition that affects everyday function (1). It is an umbrella term for a number of specific medical conditions, including Alzheimer’s disease (AD) (2), which is perhaps the most studied subtype of dementia. In 2019, the global number of individuals who lived with dementia was estimated at 57.4 million and, largely due to population growth and ageing, this figure is expected to approximately triple by 2050 to reach 152.8 million (3).

In the UK, 885,000 people were estimated to live with dementia in 2019; the majority of them (84.7%) residing in England (4). The number of people with dementia in the UK has been projected to increase to 1.6 million by 2040, including 1.35 million people in England alone (4). The economic burden of dementia in the country is substantial, with the total cost of care estimated at £34.7 billion across the UK in 2019, of which publicly or privately funded social care constituted 45% (£15.7 billion), informal care 40% (£13.9 billion), and health care 14% (£4.9 billion) (4). By 2040, the total costs of dementia care have been projected to rise 2.7-fold from the 2019 estimates, to reach approximately £94.1 billion (4).

Currently there are no disease-modifying therapies are available in the UK and the existing pharmacologic interventions work symptomatically (5, 6). In addition, non-pharmacological interventions such as cognitive stimulation, cognitive rehabilitation, and occupational therapy are recommended to promote independence and well-being in people with dementia (6). However, there are still potential benefits to diagnosing dementia early. Firstly, there is some evidence beginning to emerge that early treatment for AD delays cognitive decline (7). Secondly, early awareness of diagnosis facilitates informed decisions, e.g., related to financial and legal planning or future care needs (8). Finally, with the advent of disease-modifying therapies (DMTs), which target the pathophysiology of AD to delay its progression (9), the importance of early dementia diagnosis will be growing in years to come and early diagnosis may provide the opportunity to participate in research.

Early diagnosis of dementia at a stage where the cognitive impairment is still mild can be achieved in the primary care setting using an established cognitive assessment instrument (8, 10). In the UK, individuals with suspected dementia are subsequently referred to a memory clinic to establish the subtype of dementia based on further diagnostic testing and initiate therapy as appropriate (10). Cognitive testing is often also employed during initial triage in the memory clinic (11). The COVID-19 pandemic, however, necessitated conducting cognitive assessment remotely, despite unclear validity and reliability of such assessments using currently available tools (12).

The integrated cognitive assessment (ICA), trademarked as CognICA^™^, is a brief, language independent, self-administered, computerized cognitive test, which uses an explainable artificial intelligence model to improve its accuracy of cognitive impairment diagnosis (13–15).

The ICA is previously validated across multiple studies as an accurate cognitive assessment tool compared with clinical diagnosis (13–18) and have further demonstrated convergent validity with existing standard of care tests, such as MoCA and ACE (13–15). The tool is also CE-Marked^1^ and FDA-registered^2^ as a software-as-medical-device.

The ICA aims to facilitate and streamline dementia diagnosis in the National Health Service (NHS). In this real-world Accelerating Dementia Pathway Technologies (ADePT) study (19), the objective was to assess health economic benefits of this novel clinically validate ICA test in comparison to the current standard of care (11). Therefore the current analysis offer a comprehensive economic evaluation of the ICA tool compared with standard cognitive tests used for dementia screening in the primary care setting and for initial patient triage in the memory clinic setting.

## Methods

In the base case analysis, the ICA tool was compared to standard cognitive testing in the primary care setting, comprising the mini-mental state examination (MMSE), general practitioner assessment of cognition (GPCOG), the six-item cognitive impairment test (6CIT), the abbreviated mental test score (AMTS), the Montreal cognitive assessment (MoCA) and a small proportion received a mix of other tests. A scenario analysis was also conducted in people with suspected dementia that have been referred to a memory clinic, where the ICA tool was tested against standard care comprising the Addenbrooke’s cognitive examination-III (ACE-III) and the Montreal cognitive assessment (MoCA). Quality of life outcomes were quantified using quality-adjusted life years (QALYs). Both costs and QALYs were discounted at 3.5% *per annum* in line with National Institute for Health and Care Excellence (NICE) guidance (20). The NICE reimbursement threshold of £20,000 per QALY gained was used to assess the cost-effectiveness of the ICA tool over a lifetime horizon.

### Ethics approval

Health Research Authority and Health and Care Research Wales approval for this study was obtained in February 2020. The study is registered in the ISRCTN Registry (ISRCTN16596456). Approved 27/02/2020, North of Scotland Research Ethics Committee (Summerfield House, 2 Eday Road, Aberdeen, AB15 6RE, UK; +44 (0)1224 558458; nosres@nhs.net), ref.: 20/NS/0029.

### Model structure

**Figure 1:**
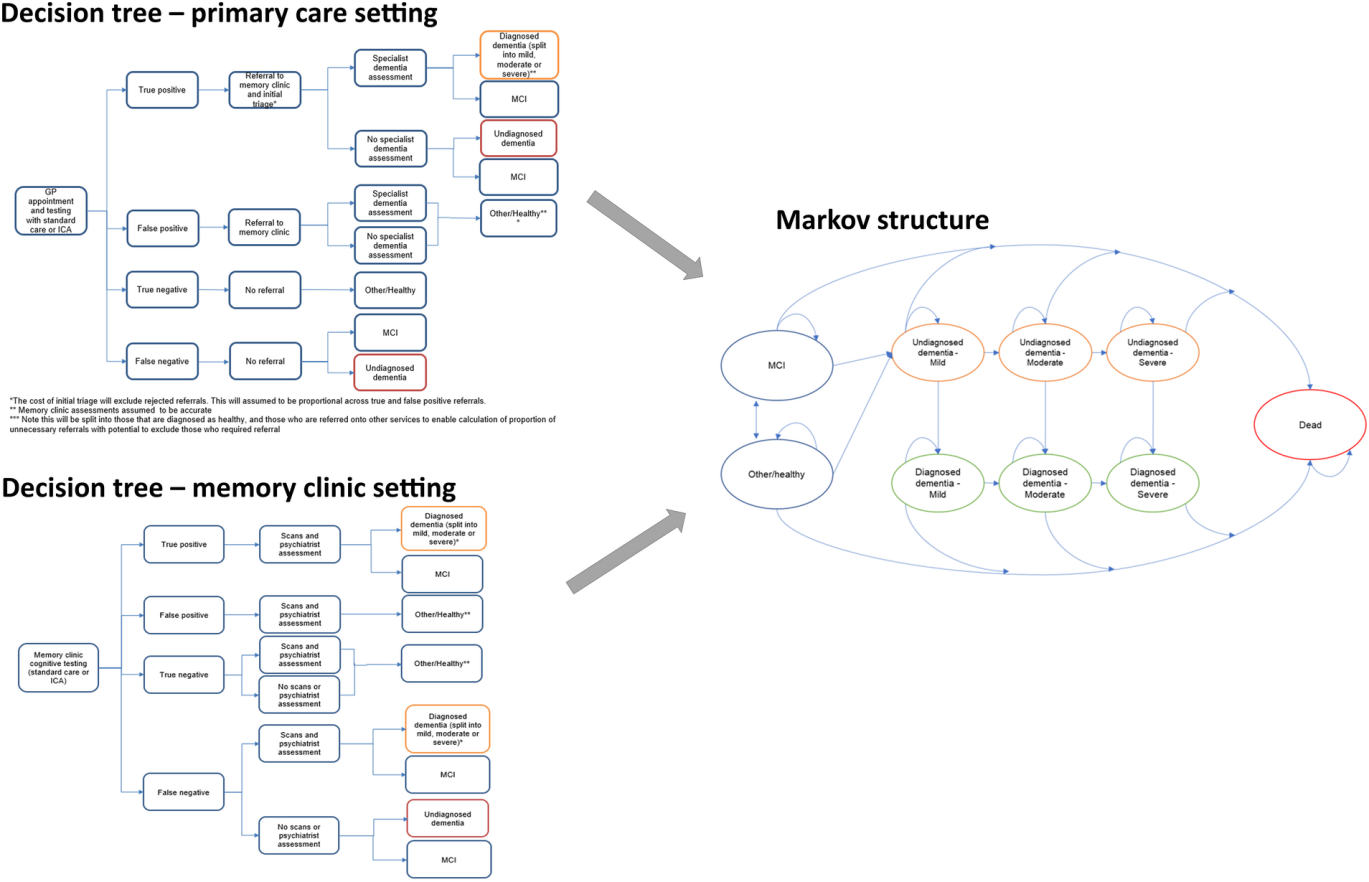
Structure of the model including the two alternative decision tree components (left) and the Markov component (right).

Model structure is summarized in Figure 1. The structure was informed by the Sussex partnership memory clinic care pathway and a targeted literature review. The model employed a decision tree to capture the initial outcomes of the cognitive testing, followed by a Markov structure to capture long-term outcomes after the initial test. A 1 year cycle length was used to capture dementia progression in the Markov model. The model includes several states such as mild cognitive impairment (MCI), diagnosed dementia, undiagnosed dementia, and Other/Healthy, along with the associated transitions from mild to moderate to severe and dead. Please refer to Figure 1 for a graphical representation of the model’s structure. The perspective of the analysis was that of the UK NHS and Personal Social Services (PSS).

### Clinical and quality of life inputs

A summary of model inputs is provided in Tables 1–7.

**Table 1:** Model inputs applied in the base case and scenario analyses: base case (primary care setting).

Parameter	Input	Source
**Epidemiology**		
Proportion of patients attending GP with symptoms	0.6%	NHS Digital (21)
Proportion of patients refusing assessment in a GP clinic	6.28%	Figures from 2018 to 2019 used so as to minimize impact of the COVID-19 pandemic
Prevalence of dementia in those attending the GP	6.4%	NHS Digital (22)
Prevalence of MCI in those attending the GP	5.5%	Ozer et al. (23)
**Testing outcomes**		
*ICA tool*		
Sensitivity for MCI	83%	Modarres et al. (19)
Specificity for MCI	80%	
Sensitivity for dementia	93%	
Specificity for dementia	80%	
**Standard care cognitive tests**		
*Mini-mental state examination (MMSE)*		
Sensitivity for MCI	51%	Tong et al. (24)
Specificity for MCI	75%	
Sensitivity for dementia	59%	
Specificity for dementia	85%	
Proportion of patients receiving test	26%	
*General practitioner assessment of cognition (GPCOG)*		
Sensitivity for MCI	52%	Tong et al. (24)
Specificity for MCI	82%	
Sensitivity for dementia	60%	
Specificity for dementia	93%	
Proportion of patients receiving test	21%	NICE (6)
*Six-item cognitive impairment test (6CIT)*		
Sensitivity for MCI	66%	Tong et al. (24)
Specificity for MCI	70%	
Sensitivity for dementia	88%	
Specificity for dementia	78%	
Proportion of patients receiving test	29%	
*Abbreviated mental test score (AMTS)*		
Sensitivity for MCI	66%	Assumption^a^ (explained below the table)
Specificity for MCI	70%	
Sensitivity for dementia	81%	Sheehan (25)
Specificity for dementia	84%	
Proportion of patients receiving test	7%	Tong et al. (24)
*Montreal cognitive assessment (MoCA)*		
Sensitivity for MCI	80%	Ciesielska et al. (26)
Specificity for MCI	81%	
Sensitivity for dementia	91%	Tsoi et al. (27)
Specificity for dementia	81%	
Proportion of patients receiving test	6%	Tong et al. (24)
*Other*		
Sensitivity for MCI	63%	Average of other tests in use
Specificity for MCI	76%	Average of other tests in use
Sensitivity for dementia	79%	Average of other tests in use
Specificity for dementia	85%	Average of other tests in use
Proportion of patients receiving test	11%	Tong et al. (24)

a Assumption about the AMT sensitivity/specificity for MCI: in the absence of robust sensitivity and specificity data for AMT in detection of MCI, we made a conservative assumption based on its similarities with the six-item cognitive impairment test (6CIT). Specifically, we assumed a sensitivity and specificity of 66% and 70%, respectively, for AMT. The test is culturally specific and has lost some of its relevance over time, as questions such as the date of the First World War and name of the monarch carry less significance in the 21st century than they did in the 20th. Given these limitations, the assumption serves as a conservative estimate in our model.

**Table 2:** Transition probabilities and mortality.

Parameter	Input	Source
**Transition probabilities and mortality**		
Relative risk of death for undiagnosed/diagnosed dementia compared with general population	1.82	Tong et al. (24) and Aldus et al. (28)
Relative risk of death for MCI compared with general population	1.00	Tong et al. (24)
Underlying dementia severity at point of testing—proportion mild	78%	Teipel et al. (29)
Underlying dementia severity at point of testing—proportion moderate	16%	
Underlying dementia severity at point of testing—proportion severe	6%	
Proportion of patients with mild undiagnosed dementia being diagnosed each year	9%	Sensitivity of standard care tests for each severity band combined with proportion going for test (24, 30)
Proportion of patients with moderate undiagnosed dementia being diagnosed each year	13%	Sensitivity of standard care tests for each severity band combined with proportion going for test Tong et al. (24) and Bradford et al. (30)
Proportion of patients with severe undiagnosed dementia being diagnosed each year	86%	
Progression of dementia from mild to moderate each year—undiagnosed	25.9%	Teipel et al. (29)
Progression of dementia from mild to moderate each year—diagnosed	16.0%	
Progression of dementia from moderate to severe each year—undiagnosed	18.7%	
Progression of dementia from moderate to severe each year—diagnosed	11.6%	
Yearly transition from MCI to mild undiagnosed dementia	4.9%	Tong et al. (24)
Yearly transition from MCI to “other/healthy”	16.0%	Tong et al. (24)
Yearly transitions from “other/healthy” to mild undiagnosed dementia—age up to 69 years	0.7%	Tong et al. (24)
Yearly transitions from “other/healthy” to mild undiagnosed dementia—age 70 to 74	1.1%	Tong et al. (24)
Yearly transitions from “other/healthy” to mild undiagnosed dementia—age 75 to 79	1.4%	Tong et al. (24)
Yearly transitions from “other/healthy” to mild undiagnosed dementia—age 80 to 84	2.2%	Tong et al. (24)
Yearly transitions from “other/healthy” to mild undiagnosed dementia—age 85+	6.3%	Tong et al. (24)
Yearly transitions from “other/healthy” to MCI—age up to 69 years	1.2%	Tong et al. (24)
Yearly transitions from “other/healthy” to MCI—age 70 to 74	1.8%	Tong et al. (24)
Yearly transitions from “other/healthy” to MCI—age 75 to 79	3.3%	Tong et al. (24)
Yearly transitions from “other/healthy” to MCI—age 80 to 84	3.2%	Tong et al. (24)
Yearly transitions from “other/healthy” to MCI—age 85+	2.3%	Tong et al. (24)

**Table 3:** Quality of life.

Parameter	Input	Source
**Quality of life**		
Utility for “healthy/other” health state	0.749 to 0.645	Kind et al. (31)
Utility decrement for MCI	−0.06	Handels et al. (32)
Utility decrements for diagnosed dementia—mild	−0.125	Orgeta et al. (33)^a^
Utility decrements for diagnosed dementia—moderate	−0.235	
Utility decrements for diagnosed dementia—severe	−0.305	Wimo et al. (34)
Utility decrements for undiagnosed dementia—mild	−0.129	Gomes et al. (35)
Utility decrements for undiagnosed dementia—moderate	−0.242	
Utility decrements for undiagnosed dementia—severe	−0.314	

a The disutility values were assumed to be the same for both males and females in our model, due to a lack of gender-specific disutility data in Orgeta et al. (33).

**Table 4:** Cost of testing.

Parameter	Input	Source
Cost of GP time (per minute)	£3.60	PSSRU.ac.uk (36)
Cost of practice nurse (per minute)	£0.62	PSSRU.ac.uk (36)
Total cost of laboratory dementia screening tests per patient	£8.31	PSSRU.ac.uk (36)
GP time taken to undertake/interpret ICA tool test (minutes)	2	Data on file
GP time taken to undertake/interpret standard care tests—weighted average (minutes)	8	Tong et al. (24), Sheehan (25), Yokomizo et al. (37), Tumas et al. (38) and Cordell et al. (39)
Practice nurse time to undertake ICA tool test (minutes)	10	Data on file
Practice nurse time to undertake standard care test—weighted average (minutes)	0	Tong et al. (24), Sheehan (25), Yokomizo et al. (37), Tumas et al. (38) and Cordell et al. (39)
ICA tool cost per test	£10	Data on file
Standard care cost per test—weighted average	£0.26	Tong et al. (24)
Total cost of ICA tool	£31.67	Calculated using above inputs
Total cost of standard care tests	£36.72	Calculated using above inputs

**Table 5:** Health state costs.

Parameter	Input value	Source
**Diagnosed dementia**		
Mild	£9,699	Alzheimer’s Society (40) and Getsios et al. (41)
Moderate	£32,418	Alzheimer’s Society (40) and Getsios et al. (41)
Severe	£34,301	Alzheimer’s Society (40) and Getsios et al. (41)
**Undiagnosed dementia**		
Mild	£8,973	Alzheimer’s Society (40) and Getsios et al. (41)
Moderate	£31,692	Alzheimer’s Society (40) and Getsios et al. (41)
Severe	£34,055	Alzheimer’s Society (40) and Getsios et al. (41)
Mild cognitive impairment (ICA tool)	£0.00	Anderson et al. (42)
Mild cognitive impairment (standard care)	£0.00	Anderson et al. (42)
Other/healthy	£0.00	Anderson et al. (42)

**Table 6:** Scenario (memory clinic setting).

Parameter	Input value	Source
**Epidemiology**		
Proportion of patients attending the GP with symptoms	0.6%	NHS Digital (21)
Referral rate to memory clinic	9.8%	Assumption
Proportion of patients declining referral to memory clinic	6.7%	Assumption
Prevalence of dementia in those attending the memory clinic	63%	Cook et al. (43)
Prevalence of MCI in those attending the memory clinic	18%	Assumption
**Testing outcomes**		
*ICA tool*		
Sensitivity for MCI	83%	Kalafatis et al. (11) and Modarres et al. (19)
Specificity for MCI	80%	Kalafatis et al. (11) and Modarres et al. (19)
Sensitivity for dementia	93%	Kalafatis et al. (11) and Modarres et al. (19)
Specificity for dementia	80%	Kalafatis et al. (11) and Modarres et al. (19)
**Standard care cognitive tests**		
*The Addenbrooke’s cognitive examination-III (ACE-III)*		
Sensitivity for MCI	75%	Beishon et al. (44)
Specificity for MCI	89%	Beishon et al. (44)
Sensitivity for dementia	94%	Beishon et al. (44)
Specificity for dementia	83%	Beishon et al. (44)
Proportion of patients receiving test	50%	Assumption
*Montreal cognitive assessment (MoCA)*		
Sensitivity for MCI	80%	Ciesielska et al. (26)
Specificity for MCI	81%	Ciesielska et al. (26)
Sensitivity for dementia	91%	Tsoi et al. (27)
Specificity for dementia	81%	Tsoi et al. (27)
Proportion of patients receiving test	50%	Assumption
*Weighted average of standard care tests used in model*		
Sensitivity for MCI	78%	Calculated using information above
Specificity for MCI	85%	Calculated using information above
Sensitivity for dementia	93%	Calculated using information above
Specificity for dementia	82%	Calculated using information above

**Table 7:** Cost of testing-memory clinic.

Parameter	Input value	Source
Cost of nurse time per minute (weighted average of bands 5 to 7)	£1.13	Assumed 80% at band 5, 10% at band 6 and 10% at band 7. PSSRU.ac.uk (36)
Nurse time taken to undertake ICA test (minutes)	10	Data on file
Nurse time taken to undertake standard care cognitive tests (minutes)—weighted average	16.25	Bradford et al. (30), Molloy et al. (45), Yokomizo et al. (37), Tumas et al. (38) and Cordell et al. (39)
ICA tool cost per test	£10	Data on file
Total cost of ICA testing	£21.27	Calculated using above inputs
Total cost of standard care cognitive testing	£18.31	Assumption
Cost of further tests and scans in a memory clinic	£961	PSSRU.ac.uk (36)

GP, general practitioner; ICA, integrated cognitive assessment; MCI: mild cognitive impairment.

Sensitivity and specificity of the ICA tool for diagnosing mild cognitive impairment (MCI) and dementia were derived from the results of the ADePT study (11, 19). The results from this study informed the performance of the ICA tool in comparison with specialist diagnosis obtained at the memory clinic, i.e., the accuracy of ICA for referral of patients to a memory clinic.

Underlying dementia severity in year 1 (i.e., at the point of testing) was based on the Teipel et al. (29) model assessing the cost-effectiveness of donepezil for AD and was assumed to be the same regardless of diagnosis status (i.e., in people with dementia who are diagnosed vs. undiagnosed). The proportions of the modelled population that were diagnosed with mild, moderate, and severe dementia in subsequent years were taken from the literature and assumed to be equal between the two testing arms. Progression of undiagnosed and diagnosed dementia was derived from Teipel et al. (29) Transition probabilities from MCI to mild undiagnosed dementia and between the “other/healthy” health state and MCI were sourced from a study by Tong et al. (24) assessing the cost-effectiveness of different cognitive screening tests for use in the primary care setting.

Mortality was modelled using UK general population data for individuals in the “healthy/other” health state and relative risks were applied for people with dementia to capture increased risk of death. The relative risk of death with dementia was assumed to be the same regardless of whether the condition was diagnosed or undiagnosed, in line with a previous study (28). People with MCI were assumed to die at the same rate as the general population, based on the study by Tong et al. (24).

Utility values were estimated using UK population norms (31) with literature-derived decrements applied to dementia health states.

In our Markov model, mortality risks are specifically tailored to the age and health state of the individuals in the cohort. Mortality rates are derived for the general population, those with mild cognitive impairment (MCI), undiagnosed dementia, and diagnosed dementia. These rates are adjusted according to the age of the cohort, starting from 77 years and extending onwards. This allows the model to capture the nuanced mortality risks associated with both the progression of cognitive decline and aging. For example, the death rate for the general population at age 77 is 3.5%, whereas for those with diagnosed or undiagnosed dementia, it is 6%. These rates change as the cohort ages, reflecting the increased mortality risk associated with older age and the progression of the disease. This nuanced approach ensures a more accurate representation of long-term outcomes.

### Cost and resource use inputs

The model was populated using epidemiological and cost and resource use data from official NHS and PSS data sources, wherever available (cost year 2019). Costs of testing included in the model comprised staff time to conduct the cognitive tests, laboratory testing, and the costs of further assessment and scans at a memory clinic, in addition to the cost of the ICA. The costs were not inflated to the current year of the analysis/publication, which may affect the accuracy of the economic evaluations. This is a limitation that will be considered in future updates to the model.

The cost of ICA comprised a one-off implementation fee of £2,000, followed by a minimum monthly fee of £1,000 for 100 tests with each test thereafter charged at £10. Implementation fees were charged per trust and annuitised in the model. Sourcing of iPads or tablets, which are required for ICA to operate, were not included assuming these would be already available at the primary care practice or memory clinic settings. Staff time for training on the use if the ICA tool was also not included in the model.

Based on the methods used by Getsios et al. (41), the costs of undiagnosed and diagnosed dementia were assumed to be the same, except for the cost of treatment which was applied only to those who had been diagnosed. This assumption can be considered conservative, since potential cost savings associated with earlier diagnosis (e.g., due to delay in admission to care homes) have been reported in the literature (46, 47). Treatment costs were based on NICE guidelines and costed using the BNF (10, 48).

### Model outputs

The following outcomes were assessed for each testing arm of the model and compared between arms: total costs per person, total QALYs per person, total number of diagnoses per modelled population, total number of unnecessary referrals per modelled population, and total number of scans/assessments at a memory clinic per modelled population. Head-to-head incremental comparison between the ICA tool and standard care was based on the incremental cost-effectiveness ratio (ICER), the incremental net health benefit (NHB); and the incremental net monetary benefit (NMB).

### Sensitivity analysis

In order to account for uncertainty around the input parameter values, one-way deterministic sensitivity analysis (DSA) was included in the model. The ranges used to vary the parameters were based on confidence intervals, or assumptions were made where these were not available. A probabilistic sensitivity analysis (PSA) was also implemented, whereby, depending on the parameter of interest, Dirichlet, gamma, lognormal or beta distributions were fitted to uncertain parameters to generate the input values for each iteration. The standard errors used to generate probabilistic values were derived from reported 95% confidence intervals wherever possible. Where this information was not available (e.g., for some costs), the standard error was assumed to be equal to 25% of the mean value. The appropriate number of iterations for PSA was determined by observing the number of iterations required for the average results to stabilize.

### Scenario analysis

For the sensitivity and specificity of ICA used for initial triage in the memory clinic, the tool was assessed in a scenario analysis assuming equivalent sensitivity and specificity as in the base case analysis, except when real-world data from deployments of the ICA in memory clinics were available. Model inputs specific to this scenario are listed in Tables 1–7 below the base case inputs.

Two additional scenarios are presented in the Supplementary material: a scenario considering the use of the ICA tool for remote initial assessment of patients with symptoms of dementia and a scenario considering the use of the ICA tool for remote monitoring of MCI patients.

## Results

### Base case model results (primary care setting)

The ICA tool dominated standard care comprising currently used cognitive tests, with an estimated NMB of £154 per person (Table 8). The use of the ICA tool in the primary care setting to facilitate preliminary diagnosis of dementia and referral to memory clinics resulted in cost savings of around £123 per person over a lifetime time horizon. This equated to a cost saving to the NHS of approximately −£37,122,839. when considering the total population in a given year who may benefit from the introduction of the ICA tool. A small incremental QALY benefit of 0.0016 per person was also estimated as a result of earlier dementia diagnosis.

**Table 8:** Summary of base case results (primary care setting).

	ICA tool	Standard care	Incremental
**Costs**			
Total costs	£7,791,733,892	£7,828,856,731	−£37,122,839
Cost per patient	£25,902	£26,026	−£123
*QALYs*			
Total QALYs	1,673,720	1,673,252	467.51
QALYs per patient	5.5640	5.5624	0.0016
**Other outcomes**			
ICER	Dominant		
NMB	£154		
NHB	0.01		
Total number of referrals	127,043	121,020	6,023
Total number of diagnoses	15,974	12,584	3,391
Number of unnecessary referrals	89,356	90,883	−1,528

ICA, integrated cognitive assessment; ICER, incremental cost effectiveness ratio; NHB, net health benefit; NMB, net monetary benefit; QALYs, quality-adjusted life-years.

Although there were additional costs associated with introducing the tool (implementation costs and test fees for ICA and increased costs to memory clinics for increased referrals), these costs were outweighed by the reduction in staff costs associated with conducting the initial cognitive tests and a reduction in dementia care costs associated with earlier diagnosis and therefore delayed progression to more severe dementia states (Table 9).

**Table 9:** Detailed cost breakdown in the primary care setting.

Cost breakdown (total costs)	ICA tool	Standard care	Incremental
Implementation costs	£57,778	£0	£57,778
Cost of initial testing	£9,528,113	£11,045,037	−£1,516,925
Cost of referral and memory clinic triage	£1,789,186	£1,704,366	£84,820
Cost of further assessments at memory clinic	£54,834,592	£48,745,905	£6,088,687
Other/healthy	£0	£0	£0
MCI	£0	£0	£0
Undiagnosed dementia mild	£1,142,544,575	£1,188,128,893	−£45,584,319
Undiagnosed dementia moderate	£1,788,197,563	£1,940,889,563	−£152,692,000
Undiagnosed dementia severe	£318,269,427	£355,334,234	−£37,064,807
Diagnosed dementia mild	£648,668,086	£579,875,947	£68,792,139
Diagnosed dementia moderate	£1,984,647,618	£1,832,211,993	£152,435,625
Diagnosed dementia severe	£1,843,196,955	£1,870,920,793	−£27,723,837
Total	£7,791,733,892	£7,828,856,731	−£37,122,839

ICA, integrated cognitive assessment; MCI, mild cognitive impairment.

### Sensitivity analysis

Detailed results of the sensitivity analysis are presented in the Supplementary Figure S1. Key cost-effectiveness drivers included the progression of undiagnosed dementia (transition from undiagnosed mild to undiagnosed moderate dementia), and the relative risk of death for people with undiagnosed dementia vs. the general population. Each of these parameters could change the direction of the results when varied alone. PSA results indicated that the probability of the ICA tool being cost-effective was 72% at the £20,000 per QALY threshold [see Supplementary Figure S3 for the cost-effectiveness acceptability curve (CEAC)].

### Scenario analysis (memory clinic setting)

When introduced as a triage tool in memory clinic services, ICA dominated standard care and NMB was estimated at £281 per person. Employing the ICA tool saved approximately £226 per person over a lifetime time horizon, which equated to an overall cost saving to the NHS of around −£6,613,614 (Table 10). Similar to the primary care setting, there was a small QALY gain of 0.0027 per person associated with earlier diagnosis of dementia.

**Table 10:** Summary of scenario analysis results (memory clinic setting).

	ICA tool	Standard care	Incremental
**Costs**			
Total costs	£3,039,648,934	£3,046,262,548	−£6,613,614
Cost per patient	£104,005	£104,231	−£226
*QALYs*			
Total QALYs	121,699	121,620	79.27
QALYs per patient	4.1641	4.1614	0.0027
**Other outcomes**			
ICER	Dominant		
NMB	£281		
NHB	0.01		
Total number of scans/assessment	24,041	22,799	1,242
Total number of diagnoses	17,676	17,031	644
Number of unnecessary referrals	1,872	1,572	300

The increase in initial costs due to the increased number of individuals being tested for dementia was outweighed by savings due to diagnosing dementia earlier and therefore delaying progression to more severe states of the disease (Table 11). The costs in the memory clinic setting were much higher compared with the primary care setting, due to substantially higher prevalence of dementia in this setting.

**Table 11:** Detailed cost breakdown in the memory clinic setting.

Cost breakdown (total costs)	ICA tool	Standard care	Incremental
Implementation costs	£57,778	£0	£57,778
Cost of triage	£621,540	£535,079	£86,460
Cost of further testing	£23,103,697	£21,910,146	£1,193,551
Other/healthy	£0	£0	£0
MCI	£0	£0	£0
Undiagnosed dementia mild	£59,465,260	£68,128,825	−£8,663,565
Undiagnosed dementia moderate	£110,749,823	£139,769,825	−£29,020,002
Undiagnosed dementia severe	£21,754,545	£28,798,927	−£7,044,382
Diagnosed dementia mild	£464,960,532	£451,886,186	£13,074,346
Diagnosed dementia moderate	£1,361,288,859	£1,332,317,582	£28,971,277
Diagnosed dementia severe	£997,646,902	£1,002,915,979	−£5,269,076
Total	£3,039,648,934	£3,046,262,548	−£6,613,614

DSA results revealed that the most influential parameter was the sensitivity of the ICA tool for diagnosing dementia in the memory clinic setting, which was varied by an arbitrary range of ±20% pending availability of the relevant data from the ADePT study. This parameter, alongside the two parameters already described as influential in the base case analysis (transition probability from undiagnosed mild dementia to undiagnosed moderate dementia and the relative risk of death for people with undiagnosed dementia vs. the general population) could alter the direction of the results when each of them was varied alone. In terms of PSA results, the probability of the ICA tool being cost-effective in the memory clinic setting was 63% at the £20,000 per QALY threshold [see Supplementary Figure S4 for the cost-effectiveness acceptability curve (CEAC)].

## Discussion

The ICA tool dominated cognitive assessment tests currently used in both the primary care and memory clinic settings. Introduction of the ICA tool was estimated to result in a lifetime cost saving to the NHS of approximately £123 per person undergoing cognitive assessment in the primary care setting and £226 per person being triaged in the memory clinic setting. Although the model predicted an increase in costs from increased referrals and specialist assessments, these were outweighed by a reduction in the staff costs associated with initial cognitive testing and a reduction in the costs of treatment and care for people with dementia achieved through earlier diagnosis. Increasing referrals and improving diagnosis rates is in line with the Department of Health national dementia strategy (49).

The potential for earlier diagnosis of dementia through the use of the ICA tool is particularly important given that the first DMTs for AD were approved in 2021 and 2023 (i.e., Aduhelm and Leqembi) by the Food and Drug Administration (FDA) (9). Furthermore, the AD pipeline appears rich with other DMT candidates likely to also receive approvals and becoming available (50). Potential introduction of cost-effective DMTs for AD and other types of dementia would further improve the case for early diagnosis of the condition and therefore increase the cost-effectiveness of more accurate screening (51, 52) using tools such as the ICA. The progression of dementia for those who have mild undiagnosed dementia in relation to those who have been diagnosed (i.e., they are assumed to be receiving treatment) is a key driver in the model and could change the direction of the results if there is no difference in progression of dementia with treatment. Therefore, further research in this area will also improve the robustness of the model results.

The results from the present study align reasonably well with previous UK-based analyses of similar decision problems published in recent years. Tong et al. (24) reported on a cost-effectiveness analysis undertaken to assess the use of different cognitive screening tests for diagnosing dementia and MCI in primary care. The results of Tong et al. (24) employing the health and social care perspective were similar to this analysis, ranging from a cost of £6.93 to a saving of £185 per person over a lifetime horizon, depending on the cognitive test used. However, the incremental QALYs reported were more optimistic than in the present analysis (24), potentially due to the differences in sources informing utility data. Getsios et al. (41) conducted an economic evaluation comparing early assessment and treatment for AD with early assessment and no treatment, as well as treatment without early assessment. Although the estimated cost savings from early assessment and treatment were much higher than that in this analysis (£2,135 per person when compared to treatment without early assessment and £3,593 when compared to no early assessment and no treatment) (41), some important differences in model design and assumptions between the Getsios et al. model and the present study could explain these differences. The Getsios et al. analysis estimated the potential savings from assessing and treating all individuals with dementia early and assumed 100% assessment accuracy, whereas this study estimated cost savings resulting from only a proportion of individuals benefiting from earlier diagnosis and treatment, i.e., those diagnosed with the ICA tool that would have not been diagnosed with standard cognitive tests. Therefore, the cost savings estimated in this analysis could be expected to be more modest.

Although the present model concerns a highly relevant issue considering the ageing UK population and the potential advent of DMTs for dementia, it should be noted that the analysis was subject to several limitations. Firstly, the ADePT study which informed inputs related to ICA accuracy did not include a comparison between ICA and other cognitive assessment instruments, although it did compare the outputs from the ICA tool with further testing undertaken in a memory clinic which can be considered the gold standard. Since the sensitivity and specificity inputs for standard cognitive tests were based on information extracted from the literature with no attempts to validate them, the current study should be considered a naïve comparison. Additionally, the ADePT study did not include participants who were not referred by their GPs to memory clinics, so its population is somewhat different than the population included in the model.

There is a paucity of data around the influence of diagnosis on the health care costs and quality of life associated with dementia. Therefore, the health care costs associated with diagnosed and undiagnosed dementia for each severity level were assumed to be the same, except for treatment costs assigned only to individuals who had received a dementia diagnosis. The potential benefits associated with earlier diagnosis of dementia have been described in the literature (8), however, no studies generalizable to the UK NHS that quantify these benefits in terms of the relative difference in health care costs between individuals with and without a diagnosis could be identified. Similarly, the quality-of-life benefits associated with diagnosis do not seem to be widely reported across prior cost-effectiveness analyses and literature reviews (41, 46, 47, 53). The base case utility value employed in the present study was based on Gomes et al. (35), who reported EQ-5D data at baseline and 6 months following diagnosis. However, 6 months after diagnosis can be considered a short time frame in a chronic condition, so employing this value would lead the model to understate quality of life benefits if quality of life gains were made past the first 6 months following diagnosis. The uncertainty associated with the cost and quality of life impact of dementia diagnosis could affect the cost-effectiveness of the ICA tool, as there were fewer individuals with undiagnosed dementia in the ICA arm of the model, so that increasing the cost or disutility applied to undiagnosed dementia would improve the cost-effectiveness of the ICA tool, while reducing these costs or utility decrements would decrease the cost-effectiveness of the tool.

The model also did not account for all potential costs. The cost of supplying iPads or tablets, which would be required for the ICA tool to be used in practice, was not included, assuming that these would be available at GP practices and/or memory clinics. In reality, most clinics and practices can be expected to have access to such devices, so that the costs of any additional purchases would likely be negligible. In addition, no costs were assigned to individuals in the MCI health states who were assumed to receive no regular monitoring or treatment in line with current treatment pathways. If such costs were included, they would be higher in the ICA tool arm of the model, so any benefits associated with monitoring would need to outweigh these additional costs.

The model employed the NHS and PSS perspective; consequently, the costs associated with informal care and other out-of-pocket costs were not included, even though much of the cost and burden of care falls on the person with dementia and their family (54). Anderson et al. (42) estimated that the costs of unpaid care and other costs falling on individuals vary between £9,711 for individuals with severe dementia to £17,917 for those with milder dementia. This equates to anywhere between approximately 25% of the total costs estimated per year for individuals with moderate and severe dementia up to 70% for those with milder dementia who may receive less formal care (42). However, there is substantial uncertainty associated with costing informal care. The aforementioned analysis by Tong et al. (24) estimated only a small change in results when informal care costs were included. It should also be noted that, in addition to informal care costs, the costs of lost productivity (for people with dementia or their caregivers) were also not considered, even though by 2040 these costs are estimated to reach £1.3 billion to businesses in England (40). If informal care costs and productivity costs were to be included in this analysis this would further improve the cost-effectiveness of the ICA tool.

Regarding the generalizability of the model, it is also worth noting that our model focuses on a specific setting and cohort. While the results provide valuable insights for that particular context, caution should be exercised when generalizing these findings to other settings or populations. We acknowledge the importance of using large and representative real-world data sets to inform and validate health-economic models. Our model incorporates data from only one study [i.e., Modarres et al. (19)], which may limit the generalizability of our findings.

Quality of life of caregivers or family members was also not considered, despite the substantial impact that caring for people with dementia has on them (55). Including these costs and quality of life benefits would likely improve the case for the adoption of the ICA tool because higher costs associated with dementia, particularly more severe states, could be expected to result in higher cost savings (provided the costs for diagnosed individuals are not substantially higher than those for undiagnosed individuals). It should be noted, however, that the cost-effectiveness threshold used in this analysis would no longer be relevant when considering the societal perspective because it is intended to represent the opportunity cost of funding an intervention using NHS and PSS budgets. Therefore, interpretation of the cost-effectiveness results is more difficult when considering this wider perspective.

A further limitation of the model is that it does not consider the wider impact of introducing the ICA tool on health care system capacity. Introducing a cognitive test that has increased sensitivity but decreased specificity compared with standard care would lead to increased number of referrals to memory clinics, thus having an impact on the clinic capacity. Similarly, introducing a test with increased sensitivity but reduced specificity in memory clinics as a triage tool would likely lead to more patients being routed to further testing. The costs of expanding the capacity of these services or any other services that may be impacted by an increase in diagnosed cases of dementia has not been considered in this analysis, nor has the impact of the likely increase in waiting times to access the services.

Finally, this model only considers the use of the ICA tool in place of current cognitive tests used in clinical practice. The ICA tool may have potential other uses including remote testing or monitoring of patients, although further clinical evidence and economic analyses would be needed to evaluate this.

## Conclusion

Introduction of the ICA tool within the NHS as a diagnostic tool for cognitive impairment and dementia in place of cognitive assessment tools currently used in primary care could be cost saving to the NHS. The potential cost savings were even greater when the tool was evaluated as a triage tool in memory clinics; future studies are needed to collect more real-world data in memory clinic settings to investigate the generalization of the results across wider populations in such settings.

### Precis

The ICA Tool is estimated to be cost-effective compared with standard cognitive assessments for dementia screening in primary care and memory clinic settings.

## Data availability statement

The original contributions presented in the study are included in the article/Supplementary material, further inquiries can be directed to the corresponding author.

## Ethics statement

The studies involving humans were approved by Approved 27/02/2020, North of Scotland Research Ethics Committee (Summerfield House, 2 Eday Road, Aberdeen, AB15 6RE, UK; +44 (0)1224 558458; nosres@nhs.net), ref.: 20/NS/0029. The studies were conducted in accordance with the local legislation and institutional requirements. The participants provided their written informed consent to participate in this study.

## Author contributions

JS developed the model and wrote the manuscript. CK, MM, and S-MK-R led the study design, commented on the model, and edit/revised the manuscript. AS helped with the revisions of the manuscript. All authors contributed to the article and approved the submitted version.

## Footnotes

^1^ https://pard.mhra.gov.uk/manufacturer-details/22053

^2^ https://www.accessdata.fda.gov/scripts/cdrh/cfdocs/cfRL/rl.cfm?lid=755440&lpcd=PTY
